# Physical Properties of Gelatin-Based Hydrogels Incorporated with Soybean Straw Nanocellulose Obtained by Enzymatic Hydrolysis

**DOI:** 10.3390/foods14132269

**Published:** 2025-06-26

**Authors:** Lía Ethel Velásquez-Castillo, Gisele Imoto de Freitas, Izabel Cristina Freitas Moraes, Milena Martelli Tosi, Daniel Enrique López Angulo, Paulo José do Amaral Sobral

**Affiliations:** 1 Department of Food Engineering, School of Animal Sciences and Food Engineering, University of São Paulo, Pirassununga 13635-900, SP, Brazil; gisele.imoto@usp.br (G.I.d.F.); bel@usp.br (I.C.F.M.); mmartelli@usp.br (M.M.T.); 2 Escuela de Nutrición, Facultad de Salud, Universidad Santo Tomás, Talca 3460000, Chile; dlopez22@santotomas.cl; 3 Food Research Center (FoRC), School of Pharmaceutical Sciences, Av. Prof. Lineu Prestes, 580 Bloco 14, São Paulo 05508-000, SP, Brazil

**Keywords:** biopolymer, nanofiber, nanocomposite, crops waste, renewable resource, food packaging

## Abstract

Gelatin hydrogels for food packaging applications have aroused interest in recent years. However, these hydrogels exhibit several limitations, such as poor mechanical strength and low swelling and water uptake. To overcome these challenges, nanocellulose can be used as a nanofiller. Thus, cellulose nanofibrils (CNFs) were obtained from soybean straw and used as a nanofiller for hydrogels produced with type A and B gelatin. The effects of the biopolymer type and the influence of CNF concentrations (0–3.0%, *w*/*w*) on the properties of hydrogels were studied. The CNFs exhibited a fiber morphology with micrometer length and nanometer diameter (16.8 ± 1.2 nm). The addition of CNFs (0–3%, *w*/*w*) caused a decrease in the stress (~50%) and elongation (~14%) at the fracture of the hydrogels for both type of gelatin. However, the elastic modulus increased (~20%). The addition of CNFs increased the hardness of the hydrogels up to 25%. The swelling capacity decreased by ~30% when the CNF concentration increased from 0 to 3%, while the thermal properties and chemical structure were not altered. These findings provide valuable insights for ongoing research into the incorporation of nanocellulose in biopolymer-based hydrogels produced by physical and sustainable methods for food packaging applications.

## 1. Introduction

Hydrogels are three-dimensional polymer matrices formed by chemical cross-linking or physical junctions that can retain a large amount of water or aqueous solutions [1]. These materials are widely used in the food industry as fat replacers and target delivery vehicles and to encapsulate agents and create complex shapes through 3D printing [2]. Recently, hydrogels based on natural biopolymers have also demonstrated significant potential for applications in food packaging systems: in their wet state as carriers of bioactive components for producing smart and active hydrogels or as coatings for modified paper-based food packaging, for instance [3,4]; and in their dry state as humidity controllers inside the packaging for preserving the sensorial attributes of packaged foods [5].

Biopolymers such as polysaccharides (e.g., pectin, starch, and chitosan) and proteins (e.g., soy protein, casein, and gelatin) have been studied for hydrogel production [1,5]. Among these biopolymers, gelatin appears an interesting raw material for hydrogel production due to its intrinsic properties, and is the second-most frequently employed hydrocolloid after starch [6].

Gelatin is a protein obtained by partial hydrolysis of collagen that can be solubilized in hot water and form a hydrogel when cooled below its gelling temperature [7]. Despite the multiple advantages offered by this natural biopolymer, gelatin-based hydrogels exhibit several limitations, including poor mechanical and thermal properties, as well as high swelling and water uptake [5]. An effective strategy to improve the properties of hydrogels based on natural biopolymers can be the incorporation of nanoparticles into its matrix. Among the conventional nanomaterials, nanocellulose has received considerable attention due to certain characteristics such as its abundance, biodegradability, high strength, and ease of surface modification [8]. This nanomaterial is commonly classified as nanocrystals and nanofibers, specifically called nanofibrils in the context of this study. Cellulose nanocrystals (CNCs) are rigid, rod-shaped structures measuring 2–20 nm in diameter and 100–500 nm in length with high crystallinity (54–88%), while cellulose nanofibrils (CNFs), are flexible fiber-shaped structures measuring 1–100 nm in diameter and 500–2000 nm in length with lower crystallinity (40–60%) [9].

Several raw materials have been studied in the production of nanocellulose. In recent years, alternative sources have been studied, with an emphasis on agricultural residues obtained from cereals and other crop processing [10,11], as they are renewable, sustainable, abundant, and low-cost resources with shorter growth cycles than wood, the main raw material in question.

In this context, soybeans are one of the most important crops in the world due to their multiple uses in human and animal nutrition and industrial processes. Currently, global soybean production has reached around 429 million metric tons and is led by Brazil, which was responsible for 169 million tons in the 2024–2025 harvest [12]. Soybean production generates 3 to 4 tons of soybean residues per hectare that are underutilized as supplementary feed for ruminants or burned [13]. This residue has an average composition of 35% cellulose, 21% insoluble lignin, 17% hemicelluloses, 11% ash, and 1% acid-soluble lignin, with small fractions of protein and pectin [14].

The production of nanocellulose from agro-industrial wastes consists mainly of two steps: (1) chemical pretreatment of the raw material, usually alkaline treatment followed by bleaching, which aims to remove undesirable components of the plant cell wall (e.g., hemicellulose, lignin, pectin, and waxes); and (2) obtaining nanocellulose by chemical (e.g., acid hydrolysis or d 2,2,6,6-tetramethylpiperidinyl-1-oxy (TEMPO)-mediated oxidation), biochemical (enzymatic hydrolysis), and/or mechanical methods (e.g., high-pressure homogenization and ultrasonication) [10,15]. At the same time, there is a growing interest in simple, economical, and scalable methods that can replace the use of chemical reagents because of human health and environmental concerns. Thus, the production of nanocellulose from soybean residues has been studied using different methods: nanocrystals from straw (steam and pods) [14] and from hulls or pods [16] were obtained by acid hydrolysis followed by ultrasonication; straw nanofibrils were produced by enzymatic hydrolysis followed by ultrasonication [14,17]; and nanofibrils from soybean residues were obtained by high-pressure homogenization [18,19].

Considering the growing interest and demand in the development of packaging based on natural biopolymers with enhanced mechanical and thermal properties [20], soybean straw nanocelluloses were selected as a reinforcement material to be applied in hydrogels.

Few studies on natural biopolymer-based hydrogels reinforced with nanomaterials for food applications, such as the incorporation of chitin nanowhiskers and nanofibrils into gelatin matrices [21,22] or nanofibrils and nanocrystals into pectin matrices [23], were found in the literature. The majority of research has been in the biomedical area, with particular interest in drug release-controlled systems [24,25] and to remove pollutants from water [26]. According to Mushtaq et al. [6], there is a need for further studies to critically summarize the applicability of gelatin hydrogel not only in biomedical applications but also in other environmental and technical sectors. Therefore, the main objectives of this study were to develop hydrogels based on gelatin-containing CNFs from soybean straw and to evaluate the effects of the gelatin type (A or B) and the CNF concentration on the main physical properties of the material.

## 2. Materials and Methods

### 2.1. Materials

Soybean straws were supplied by Embrapa Soja (Londrina, PR, Brazil). The enzyme cocktail Optimash™ VR was donated by DuPont Inc. (Newark, NJ, USA). Type A pigskin gelatin (molecular weight ~52 kDa; bloom 260) was purchased from Gelnex (Itaiópolis, SC, Brazil), type B bovine gelatin (molecular weight ~45 kDa; bloom 250) was donated by Gelco (Pedreira, São Paulo, Brazil), and glycerol was purchased from Labsynth Company (São Paulo, Brazil). Hydrogen peroxide (H_2_O_2_) was purchased from Merck Millipore Corporation, and magnesium sulfate heptahydrate (MgSO_4_·7H_2_O), sodium hydroxide (NaOH), acetone and ethanol (PA) were acquired from Dinâmica Química Contemporânea LTDA (Indaiatuba, SP, Brazil).

### 2.2. Production of Soybean Straw Nanofibrils

First, it was necessary to chemically treat the soybean straw (SS), which consisted of stems and pods at a 30:70 ratio, to remove non-cellulose compounds as per Martelli-Tosi et al. [27]. Briefly, 100 g of milled soybean straw was mixed with a solution of 17.5% NaOH (*w*/*v*), at a 1:10 ratio (*w*/*v*) with magnetic stirring at room temperature (20–30 °C) for 15 h. Subsequently, the suspension was filtered through 100- and 270-mesh sieves (0.150 and 0.053 mm) and washed with distilled water until neutrality. The resulting material was bleached with a blend of 2% NaOH (*w*/*v*), 4% H_2_O_2_ (*w*/*v*) and 0.3% MgSO_4_·7H_2_O at 90 °C for 3 h. This suspension was cooled and filtered through sieves, and the retained fibers were washed with distilled water until reaching neutrality and finally rinsed with ethanol and acetone. This material, called treated soybean straw (TSS), was dried in an oven with forced air circulation at 50 °C for 72 h (MA 035, Marconi, São Paulo, Brazil), passed through a 28-mesh sieve (0.589 mm), and stored at room temperature until it was used for cellulose nanofibril production.

Cellulose nanofibrils (CNFs) were produced by an enzymatic method as per Martelli-Tosi et al. [17] with slight modifications. A mass of 3 g of TSS was mixed with 150 mL of sodium acetate solution (pH 4.0) and 280 µL of the enzyme cocktail Optimash™ VR (DuPont Inc., Wilmington, DE, USA). This mixture was kept at 50 °C with constant mechanical stirring at 120 rpm for 42 h. After this period, the reaction was stopped by denaturing the enzymes at 96 °C for 5 min. This CNFs contained in the acetate solution appeared as a whitish material and were separated from the acetate solution and then resuspended in distilled water.

Subsequently, the suspensions with nanofibrils were homogenized using an UltraTurrax (Ultraturrax^®^ IKA T25, Labotechnik, Staufen, Germany) at 15,000 rpm for 5 min and sonicated using a probe ultrasound (Sonifier^®^ SFX550, Branson Ultrasonics, Brookfield, CT, USA) at 550 W and 20 kHz at 70% amplitude for 3 min. Finally, CNF dispersions were freeze-dried (FD 1.0–60E, Heto-Holten A/S, Allerod, Denmark). The nanofibril production yield was calculated as the percentage ratio between the dry mass of obtained nanofibrils and the dry mass of the soybean straw.

### 2.3. Production of Gelatin Hydrogels Containing Cellulose Nanofibrils

Gelatin hydrogels were prepared with type A or B gelatin without any previous treatment and nanofibril suspensions (0, 0.25, 0.5, 1.0, 3.0 g/100 g biopolymer) [25]. Nanofibril suspensions were prepared from freeze-dried material resuspended at the mentioned concentrations in distilled water and homogenized with Ultra Turrax (Ultraturrax^®^ IKA T25, Labotechnik, Staufen, Germany) at 15,000 rpm for 5 min and probe ultrasound (Sonifier^®^ SFX550, Branson Ultrasonics, Brookfield, CT, USA) at 70% amplitude for 3 min 24 h before use [22].

The gelatin hydrogel preparation was the same for both types. Type A or B gelatin (10 g/100 g solution) was dispersed conveniently in the nanocellulose suspension at room temperature for 30 min under magnetic stirring at 300 rpm (AA-2050, Gehaka, São Paulo, Brazil) at 60 °C for 30 min [21]. Then, these solutions were cooled at 40 °C, placed in molds, and stored at 4 °C for 24 h to hydrogel setting in a wet state [22]. Hydrogels in a dry state were also obtained for some characterizations. These materials were frozen in liquid nitrogen, freeze-dried (FD 1.0–60E, Heto-Holten A/S, Allerod, Denmark), and stored over silica gel at 25 °C for 7 days.

### 2.4. Characterization of the Soybean Straw and the Cellulose Nanofibrils

#### 2.4.1. Chemical Composition of Soybean Straw

The chemical composition of the soybean straw was determined before and after chemical treatment regarding its moisture content and mineral content as per Silva and Queiroz [28]. The cellulose, hemicellulose, and lignin content was determined by obtaining neutral detergent fiber (NDF) and acid detergent fiber (ADF) as per Van Soest [29].

#### 2.4.2. Morphology

The morphology of SS and TSS was evaluated by scanning electron microscopy (TM3000, Hitachi, Tokyo, Japan). Samples were placed on an aluminum support with double-sided carbon tape and analyzed under an accelerating voltage of 15 kV. This equipment did not demand sample metallization.

For AFM analysis, nanofibril suspensions (0.01%, *w*/*v*) were homogenized in Ultraturrax (Ultraturrax^®^ IKA T25, Labotechnik, Staufen, Germany) at 15,000 rpm for 5 min. Then, 5 mL of this nanofibril suspension was applied on a mica sheet, dried at room temperature, and analyzed using atomic force microscopy (NT-MDT, Moscow, Russia) in semi-contact mode with a resonance frequency of 150 kHz, contact force of 5 N/m, and scan speed of 0.4 Hz. The diameters of the nanofibrils were determined by line-height profile analysis using Solver Next software [11].

#### 2.4.3. X-Ray Diffraction

X-ray diffraction patterns of SS before and after chemical treatment and of CNFs were obtained using an X-ray diffractometer (Miniflex600, Rigaku, Tokyo, Japan) operating at 40 kV and 15 mA (Cukα radiation, *λ* = 1.54056 Å). Samples were analyzed in the step-scan mode in a range from 2θ = 4 to 40° at 2° min^−1^ [27]. Crystallinity index (CI) was calculated using Equation (1) [30].

(1) $$CI=\left(\frac{I_{002}-I_{am}}{I_{002}}\right)\times 100$$

where *I*_002_ is the intensity of the 002 lattice diffraction at 2θ = 22.8° (crystalline contribution) and e *I_am_* is the intensity of the diffraction at 2θ = 18° (amorphous contribution).

#### 2.4.4. Zeta Potential

The zeta potential of CNFs was measured using a ZetaPlus equipment (Brookhaven Instruments Corp., Holtsville, NY, USA). Samples were prepared in deionized water (0.01%, *w*/*v*) and analyzed using an electrolyte solution of 1 mM KCl at 25 °C [31,32].

#### 2.4.5. Chemical Structure

The chemical structure of SS, TSS, and CNFs was studied using a Fourier transform infrared spectrometer (Spectrum-One, PerkinElmer Inc., Waltham, MA, USA) with a universal attenuated total reflectance accessory. Data collection was carried out in a region of 600 to 4000 cm^−1^ at a resolution of 4 cm^−1^, acquiring 20 scans for each sample [14].

### 2.5. Characterization of Gelatin Hydrogels Containing Cellulose Nanofibrils

#### 2.5.1. Visual Aspects

The hydrogels were evaluated subjectively and are described in terms of visual appearance.

#### 2.5.2. Color and Opacity

The color and opacity of the hydrogels containing the nanofibrils were measured in samples of 1.5 mm height and 90 mm in diameter [22] using a colorimeter (Miniscan MSEZ 1049, HunterLab, Reston, VA, USA) with illuminant D65 and an angle of 10°. CIELab color space, luminance (L*) ranging from 0 (black) to 100 (white), a* ranging from green (−a) to red (+a), and b* ranging from blue (−b) to yellow (+b), was used to characterize color. The samples were analyzed on the surface of a white standard (L*_standard_ = 94.51 ± 0.03, a*_standard_ = −0.76 ± 0.01, b*_standard_ = 2.07 ± 0.01). The total color difference (∆E*) was calculated with Equation (2):

(2) $$∆E*=\sqrt{{\left(∆L*\right)}^{2}+{{\left(∆a*\right)}^{2}+\left(∆b*\right)}^{2}}$$

where ΔL* = L*_sample_ − L*_standard_; Δa* = a*_sample_ − a*_standard_; and Δb* = b*_sample_ − b*_standard_.

The hydrogel opacity was determined with the Hunterlab method in reflectance mode using the colorimeter described above and calculated as a ratio (Y = Y_b_/Y_w_) of hydrogel opacity when placed onto black (Y_b_) and white (Y_w_) standard plates.

#### 2.5.3. Microstructure

The microstructure of the dried hydrogels containing the nanofibrils was observed using the scanning electron microscope described in Section 2.4.2.

#### 2.5.4. X-Ray Diffraction Pattern

The crystalline/amorphous structure of the dried hydrogels containing the nanofibrils was analyzed using the equipment described in Section 2.4.3. The sample was analyzed from 5 to 60° (2θ) at 2° min^−1^.

#### 2.5.5. Mechanical and Viscoelastic Properties

The mechanical and viscoelastic properties of the hydrogels containing the nanofibrils were analyzed by uniaxial compression tests and texture profile analysis and stress relaxation tests, respectively. In these tests, six cylindrical samples (20 mm × 20 mm) were analyzed using a texturometer (TA.XT2i, Stable Micro Systems, Surrey, England, UK) at room temperature (~22 °C).

For the uniaxial compression tests, cylindrical samples were compressed to a deformation of 90% at a speed of 1.0 mm·s^−1^ [21]. The stress and strain at fracture, as well as the elastic modulus, were calculated from the mechanical curves obtained using Exponent Lite Express 6.1.13.0 software (TA.XT2i, Stable Micro Systems, Surrey, England, UK). For the texture profile analysis, the samples were compressed twice to a strain of 20% (verified previously as being into the reversibility domain) with a trigger force of 5 g and 5 s between the compressions. The pre-speed, operating speed, and post-speed were 1.0 mm·s^−1^ [22]. The textural parameters recorded were hardness (g), springiness, cohesiveness, gumminess (g), chewiness (g), and resilience.

In the stress relaxation tests, the samples were subjected to a constant deformation of 20%, which was maintained for 5 min [33]. The computer registered the stress, which decreased over time, necessary for the maintenance of this deformation. The value of deformation was chosen to guarantee that the behavior of the material was within the linear viscoelastic domain. The viscoelastic properties (relaxation modulus and viscosity) were determined by fitting the Maxwell model (Equation (3)) to stress–time data [34] using exponent Lite Express 6.1.13.0 software (TA.XT2i, Stable Micro Systems, Surrey, England, UK):

(3) $$\sigma \left(t\right)=G\;\gamma_{0}e^{-t/\lambda }$$

where *σ* is stress (MPa); *γ*_0_ is constant strain (%); *G* is the relaxation modulus (MPa); *t* is experimental time (s); and *λ* is the relaxation time in terms of a viscosity (μ) (10^6^ × Pa·s^−1^) and relaxation modulus (*λ* = μ/*G*).

#### 2.5.6. Swelling and Water Retention Capacity

The swelling (SW) and water retention capacity (WRC) of the hydrogels were determined gravimetrically using hydrogel samples in a dry state. The previously weighed sample (*W*_0_) was immersed in distilled water (50 mL) for 24 h at 25 °C. After this period, the sample was removed, wiped gently to remove excess water from the surface, and then weighed (*W_t_*). The SW was calculated using Equation (4) [35].

(4) $$SW=\left(\frac{W_{t}-W_{0}}{W_{0}}\right)\times 100$$

The WRC was evaluated by saturating the weighed hydrogel samples with distilled water (*W_i_*). Then, the samples were placed in desiccators containing a saturated NaBr solution (58% RH) at 25 °C for 24 h. Subsequently, the samples were weighed (*W_r_*), and the WRC was calculated with Equation (5) [36].

(5) $$WRC=\left(\frac{W_{i}}{W_{r}}\right)\times 100$$

#### 2.5.7. Thermal Properties

The thermal properties of the dried hydrogels were analyzed by differential scanning calorimetry (DSC T2010, TA Instruments, New Castle, DE, USA). Powder samples (~5 mg) were placed in aluminum pans and analyzed from −50 to 150 °C at 5 °C/min with double scanning (heating and cooling always after quench cooling with liquid nitrogen) using an empty pan as reference. The glass transition temperature (T_g_), melting temperature (T_m_) and melting enthalpy (ΔH_m_) were calculated from heat-flow curves using Universal Analysis V1.7F software (TA Instruments, New Castle, DE, USA) [37].

#### 2.5.8. Chemical Structure

The FTIR spectra of the dried hydrogels containing the nanofibrils were analyzed using the equipment described in Section 2.4.5. Samples were analyzed in the spectral range 650–4000 cm^−1^ at 4 cm^−1^, with 20 scans for each sample [21,35].

#### 2.5.9. Statistical Analysis

All the results are presented as means ± standard deviation of tests carried out in triplicates. The data were subjected to statistical analysis by ANOVA and Tukey’s test (*p* ≤ 0.05) to verify significant differences between the means using the Statistical Analysis System (SAS) program (9.4, SAS Institute Inc., Cary, NC, USA).

## 3. Results

### 3.1. Characterization of Soybean Straw and Cellulose Nanofibrils

The alkaline treatment carried out on the SS provided a yield of 37.8 ± 1.6 g of TSS/100 g of SS. This value was lower than that reported for soybean hulls treated with alkali (46%) by Neto et al. [38], and (64.5%) by Barros et al. [39]. These differences may be related to the breakdown of cellulose chains during bleaching and the loss of material inherent to the different filtration processes. However, the yield obtained here was similar to that obtained for rice husks (36%) treated by other chemical methods by Lu and Hshieh [40].

The TSS had a lighter color (yellowish) when compared to SS (brown) (Figure S1, see Supplementary File), which indicates that parts of the initial non-cellulosic components (hemicelluloses, lignin, pectin, and proteins) were removed by chemical treatment.

#### 3.1.1. Chemical Composition of Soybean Straw

The dry matter content of SS before and after treatment increased (*p* ≤ 0.05) from 89.6 ± 0.2% (SS) to 92.0 ± 0.0% (TSS), whereas the ash content decreased (*p* ≤ 0.05) from 4.3 ± 0.0% (SS) to 1.2 ± 0.0% (TSS), indicating that the alkaline treatment managed to remove non-cellulosic components during the process. These values were close to those found in the literature for dry soybean hulls, which contained ~8% of moisture and 4.2–5.3% of ash content [39,41].

The cellulose content increased (*p* ≤ 0.05) from 41.4 ± 0.1% (SS) to 62.9 ± 0.1% (TSS) after alkaline treatment, while the hemicellulose content decreased (*p* ≤ 0.05) from 19.0 ± 0.2% (SS) to 8.9 ± 0.4% (TSS), which is related to the cleavage of substances linked to the ester groups under basic conditions [31]. This result was similar to that reported by Martelli-Tosi et al. [27], who observed an increase in cellulose from 40% to 66% and a decrease in hemicellulose from 23% to 9.5% when the same chemical alkali treatment was applied to soybean straw. In turn, Neto et al. [38] also observed that cellulose content values ranged from 48% to 85% and hemicellulose content from 24% to 11% for soybean hulls after alkaline treatment followed by bleaching with aqueous sodium chlorite.

However, the lignin content increased from 7.8 ± 0.1% (SS) to 19.4 ± 0.0% (TSS) after alkali treatment, which was unexpected, as different authors have reported that alkali treatment followed by a bleaching step with H_2_O_2_ provoked a reduction in insoluble lignin from 10.5% to 3.5% in soybean straw [27], and from 5.8% to 3.7% in soybean hulls [38]. Quite possibly, the alkaline treatment carried out on soybean straw reduces the basis for calculating the percentage of lignin, although the results suggest the presence of residual lignin in the treated soybean straw.

#### 3.1.2. Yield and Zeta Potential of CNFs

The CNFs were obtained with a yield of 10.1 ± 0.2%, which was comparable to that obtained by enzymatic hydrolysis of soybean straw (13.3%) by Martelli-Tosi et al. [14] and banana peels (10%) by Tibolla et al. [42]. Nevertheless, yields of up to 34.1% have been determined for hulled soybean residue nanofibrils (mainly cotyledons) produced by chemical treatment followed by high-pressure homogenization [19].

The zeta potential of CNFs was −28.6 ± 7.6 mV, suggesting that they can form stable colloidal suspensions [32]. The negative value of the zeta potential was associated with the ionic modification induced by the ions provided by the buffer or due to the influence of pH [14]. This value agreed with those reported for CNFs from banana peel (−25 mV) [42,43] and from soybean straw (−24.5 mV) [14] produced by enzymatic hydrolysis and CNFs from soybean residue produced by high-pressure homogenization (−22.5 mV) [19].

#### 3.1.3. Morphology

**Figure 1:**
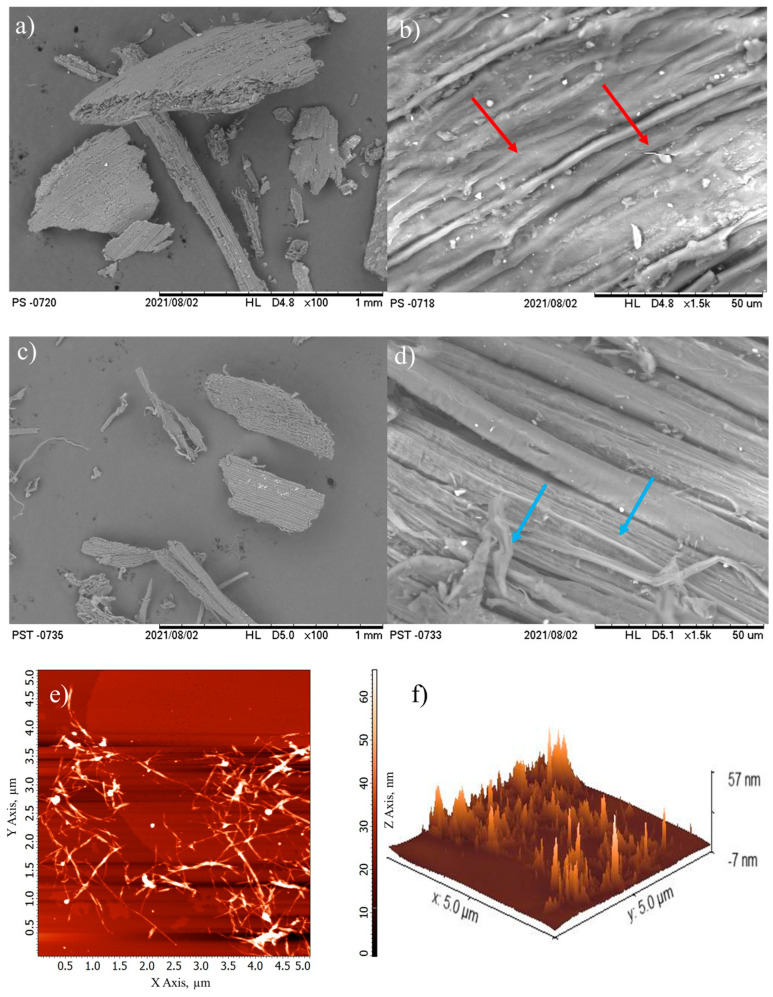
Scanning electron micrographs of (**a**,**b**) soybean straw and (**c**,**d**) treated soybean straw, and (**e**) 2D and (**f**) 3D atomic force micrographs of cellulose nanofibrils.

Scanning electron micrographs showed that SS appears as irregularly shaped compact structures with a wide range of lengths from ~50 to ~1500 µm (Figure 1a). It was also possible to observe that the SS cell wall was composed of fibers containing cellulose microfibrils organized in a parallel manner and agglomerated by lignin, as indicated by the red arrows in the Figure 1b. After alkaline treatment, soybean straw appeared as porous structures with a smooth surface and lengths ranging from ~30 to ~1000 µm (Figure 1c), in which cellulose microfibrils were clearly exposed and isolated in some cases, as evidenced by the blue arrows in the Figure 1d. This suggested that there were fewer connections in the plant structure of chemically treated soybean straw. This result was related to the partial removal of the non-cellulosic outer layer of the plant wall, which is composed of hemicellulose, lignin, and pectin [9,31].

AFM evidenced that CNFs appeared as agglomerated fibers with nanometric diameters (Figure 1e,f). Image analysis of the line height profiles allowed us to estimate that the length varied from 460 to 2000 nm, with an average diameter of 16.8 ± 1.2 nm. It is important to mention that it was not possible to calculate the length accurately due to the agglomeration observed in this type of nanocellulose. Similar observations were reported for commercial CNFs with diameter values of ~7 nm and lengths of several micrometers [44]. CNFs from microcrystalline cellulose exhibited fibril web-like structures highly entangled with each other, with an average width of ~47 nm [45]. CNFs from soybean straw presented an average diameter of 9.4 ± 3.3 nm with non-uniform lengths, and were arranged as agglomerates as per Martelli-Tosi et al. [14].

#### 3.1.4. X-Ray Diffraction Patterns and Relative Crystallinity

**Figure 2:**
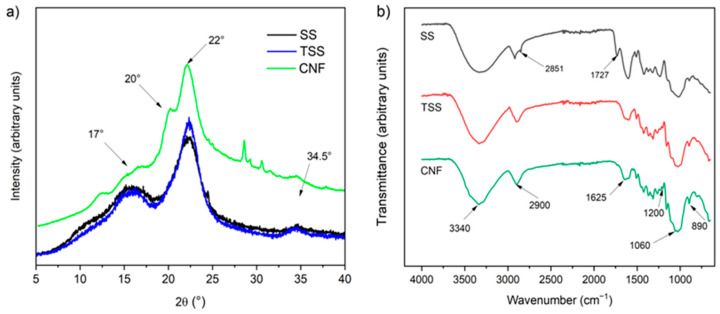
(**a**) X-ray diffraction patterns and (**b**) FTIR spectra of soybean straw (SS), treated soybean straw (TSS), and cellulose nanofibrils (CNFs).

SS, TSS, and CNFs presented broad peaks with high intensities at 2θ = 21.9°, 17°, and 34.5° (Figure 2a), which are typical of native type I cellulose [38]. For TSS, these peaks were broader and more defined, suggesting that it contained a lower amount of amorphous material (residual lignin and hemicellulose). Similar observations have been reported for cellulosic material extracted from crop residues, such as soybean hulls [19,39], corn [46], and garlic straw [31]. In addition, CNFs presented peaks at 2θ = 12.3° and 20.1° (Figure 2a), evidencing the presence of type II cellulose, which arises because of the effects of twisting and binding of fibers during alkaline treatment. Nevertheless, these peaks could not be identified for TSS (Figure 2a), probably due to lower intensity in the sample.

The crystallinity indices of the SS, TSS, and CNF samples were 43.8%, 48.3%, and 56.9%, respectively. The TSS crystallinity evidenced an increase in the proportion of crystalline cellulose because of the removal of and reduction in amorphous non-cellulosic compounds during alkaline treatment of SS [38]. Similar observations were made for soybean residues subjected to alkali treatment, such as straw (58% to 62%) [27], pods (72.9% to 81.7%) [16] and hulls (21.9% to 36.9%) [19]. On the other hand, CNFs showed a crystallinity similar to that reported for CNFs from soybean straw (45–50%) [14,17] and banana peel (49.2%) [42]. However, CNFs have been produced with relatively high crystallinity levels from soybean residue (75.9%) [19] and banana peel (66.2%) [43], higher values that could be related to a cellulose-rich soybean residue and the use of a specific enzyme (xylanase) in the hydrolysis, respectively.

#### 3.1.5. Chemical Structure

SS, TSS, and CNF FTIR spectra showed characteristic absorption bands of lignocellulosic materials (Figure 2b). Bands located at 3600–3000 and 2900 cm^−1^ corresponded to the stretching vibration of O–H groups present between cellulose and bound water and the C–H stretching of cellulose [19]. The multiple bands between 1420 and 1162 cm^−1^ were related to the typical vibrations of the primary and secondary hydroxyl bending in cellulose [27]. An intense band centered at 1030 cm^−1^ was attributed to the asymmetric vibration of C–H and the extension of C–O to the β-glycosidic bonds between β-D-glucopyranosyl units in cellulose [27]. Indeed, peaks at 1060 and 895 cm^−1^ were associated with the C–O and C–H stretching vibrations of cellulose [14].

Likewise, analyzing the CNF and TSS spectra, it was observed that the peaks at 2851 and 1727 cm^−1^ disappeared when compared to SS. The first peak was characteristic of the hemicellulose spectrum, and the second one was attributed to the acetyl and uronic ester groups of hemicellulose or to the ester bond of the carboxylic group of the ferulic and p-coumaric acids from lignin and/or hemicellulose [19,39]. This disappearance was provoked by the removal of hemicellulose and lignin during chemical treatment of SS. In turn, the CNF and TSS spectra evidenced a decrease in the intensity of the peak at 1625 cm^−1^, attributed to the aromatic structural vibration of lignin, which would indicate the residual presence of this component after the chemical treatment and enzymatic hydrolysis [38].

### 3.2. Characteristics of Gelatin Hydrogels Containing Soybean Straw Cellulose Nanofibrils

#### 3.2.1. Visual Aspect

In general, the hydrogels appeared as homogeneous materials with a characteristic color depending on the type of gelatin (Figure S2). Those produced with type A gelatin were colorless, and those produced with type B gelatin were slightly yellowish. The presence of CNFs was not visible, suggesting that they were well dispersed and homogeneous in the hydrogel matrix. When the concentration of nanofibrils increased from 0 to 3%, the hydrogels showed a notable visual change related to the increase in their opacity, as explained later.

#### 3.2.2. Color and Opacity

Type A and B gelatin hydrogels had a light tone (L* > 85), and increasing the CNF concentration did not influence this characteristic of the hydrogels (Table 1). Regarding the parameter a*, it was observed that type B gelatin hydrogels (BGHs) were greener than type A gelatin hydrogels (AGHs) (Table 1). This behavior was more notable as the CNF concentration increased from 0 to 3% for AGHs (−0.8 to −1.5) and BGHs (−1.3 to −1.8).

The b* parameter indicated that the BGHs were more yellowish than the AGHs (Table 1), which was expected because bovine gelatin has this tone. This parameter progressively increased in AGH when the CNF concentration increased from 0 to 3% (4.2 to 7.4), a result that may be related to the yellowish color of this nanomaterial. In BGH, a significant effect was observed only at the highest concentrations of CNFs (1% and 3%). The ∆E* values of the AGH and BGH were of 8.4 and 10.8, respectively, showing that these materials had a certain color. Moreover, the addition of CNFs in low concentrations (from 0.25 to 1%) did not change the color parameters of the AGH or BGH, i.e., significant differences were observed only at concentrations of 3% CNFs (Table 1).

Wang et al. [22] reported that the addition of CNFs (0–7.5%) in bovine gelatin hydrogels resulted in a slight decrease in the parameters L* (35.4–34.8) and b* (3.2–1.8) and an increase in the parameter a* (0.3–0.7). According to these authors, this color difference may be related to the birefringence closely associated with the anisotropy of these nanomaterials, as well as the light absorption of CNF aggregates and the more compact gel structure caused by the addition of CNFs.

The opacities of the AGH and BGH were ~2.4 and ~3.3, respectively, meaning that both hydrogels were almost translucent, as can be observed visually (Figure S2). These values increased when the CNF concentration was raised from 0 to 3%, a behavior that may be related to the micrometric length of this type of nanocellulose, as detailed previously. According to Althues et al. [47], nanoparticles with a diameter less than 40 nm are required to obtain transparent nanocomposites.

#### 3.2.3. Microstructure

**Figure 3:**
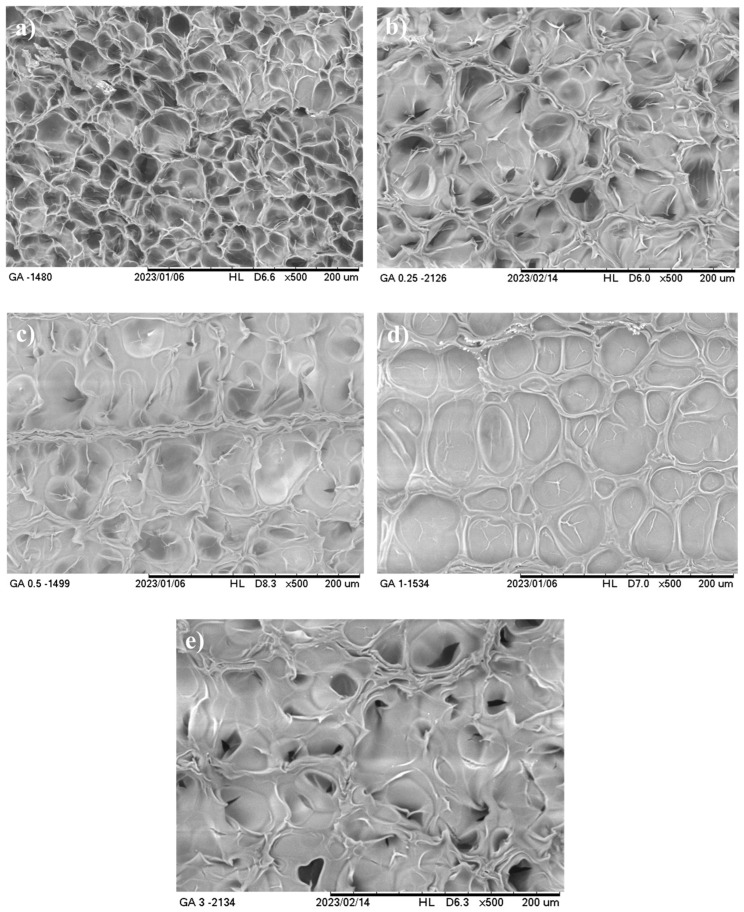
Scanning electron micrographs of type A gelatin hydrogels containing cellulose nanofibrils at (**a**) 0%; (**b**) 0.25%; (**c**) 0.5%; (**d**) 1%; and (**e**) 3%.

**Figure 4:**
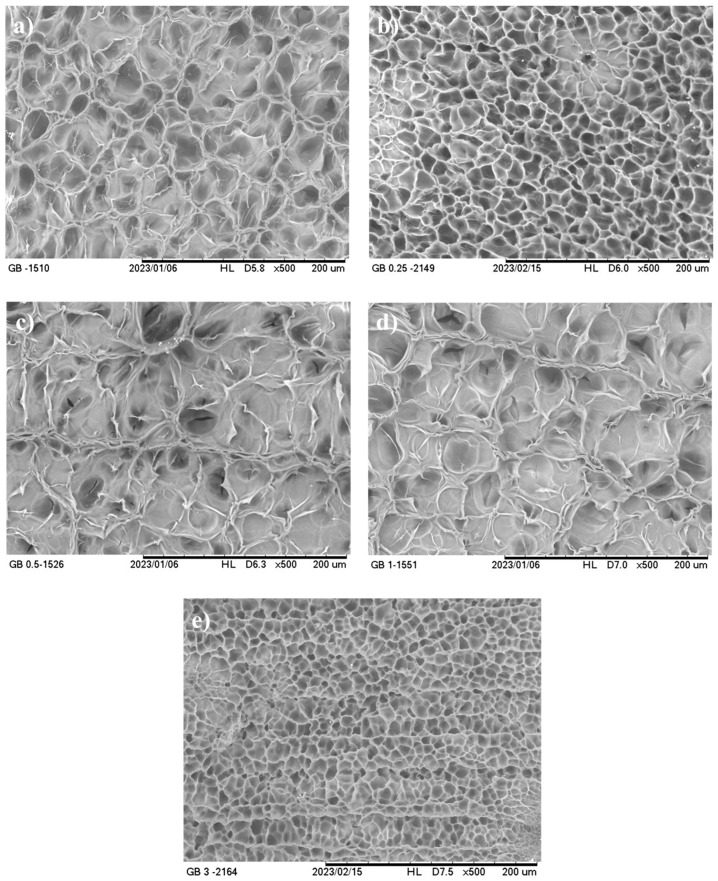
Scanning electron micrographs of type B gelatin hydrogels containing cellulose nanofibrils at (**a**) 0%; (**b**) 0.25%; (**c**) 0.5%; (**d**) 1%; and (**e**) 3%.

Dried gelatin hydrogels without CNFs appeared as three-dimensional porous structures with smooth-walled polygonal pores forming a symmetric network, regardless of the type of gelatin (Figure 3a and Figure 4a). An increase in the CNF concentration from 0 to 3% resulted in notable changes in the structure of this material (Figure 3 and Figure 4). Thus, these hydrogels presented a more compact structure, forming a less organized network, with different pore sizes as the CNF concentration increased. This effect was more evident in AGH reinforced with CNFs at a concentration of 3% (Figure 3e), which showed greater heterogeneity in the pores because of the formation of the interlocking network between the CNFs and the gelatin and/or a possible aggregation of the CNFs. For BGH at a concentration of 3% (Figure 4e), this effect did not remain, suggesting better exfoliation of CNFs in the matrix.

Similar observations were reported for gelatin hydrogels reinforced with cellulose nanofibrils (0–7.5%) [22,48] and with chitin nanowhiskers (0–1%) [21]. The addition of these nanomaterials resulted in more compact structures with smaller pores, which probably resulted in larger junction points between the gelatin and the nanomaterial and affected the size, structure, and distribution of the pores [21,22,48]. In turn, Rao et al. [48] also observed that the addition of cellulose nanofibrils to gelatin hydrogel without a cross-linking agent led to the formation of hydrogels with larger pores due to weak reversible non-covalent interactions (mainly hydrogen bonds) between cellulose nanofibrils and gelatin.

#### 3.2.4. Chemical Structure

**Figure 5:**
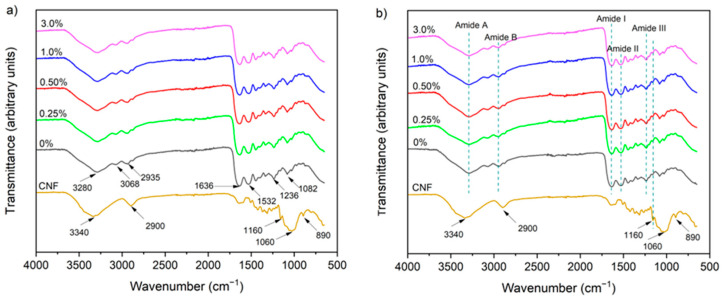
FTIR spectra of (**a**) type A and (**b**) type B gelatin hydrogels.

The spectra of the dried type A and B gelatin hydrogels were characterized by broad absorption bands centered on 3289 cm^−1^ and 2960 cm^−1^, attributed to the stretching vibration of the O–H/N–H and C–H bonds, respectively [24] (Figure 5). Characteristic bands of the amide group were also observed at 1627 cm^−1^ (amide I), 1538 cm^−1^ (amide II) and 1238 cm^−1^ (amide III), which are related to the C=O stretching vibration, N–H bending, and C–N stretching, respectively [49]. The absorption bands at 1450 cm^−1^, 1336 cm^−1^ and 1158 cm^−1^ corresponded to C–H bond bending, C–N and C stretching, and C–O bending, respectively [24].

The presence of CNFs did not alter the absorption peaks of the hydrogels, regardless of the type of gelatin, a behavior that may be related to the low CNF content and the non-use of cross-linking promoters. In other words, physical interactions between CNFs and gelatin would not be sufficient to alter the polypeptide matrix. However, the increase in band intensity at 1160 cm^−1^ in gelatin hydrogels (Figure 5) could be related to stretching of the C–O–C group of cellulose [49].

Rao et al. [48] added amino-functionalized CNFs (5%, *w*/*w*) to gelatin hydrogels and observed a shift in the OH/NH stretching vibration band, which was related to the formation of new hydrogen bond interactions between CNFs and the gelatin. In turn, Boughriba et al. [49] added cellulose microcrystals (5% to 25%) to gelatin hydrogel and observed a slight shift in the amide I band, which they attributed to the disruption of hydrogen interactions in the carbonyl groups of gelatin by competitive binding of crystalline cellulose. Thus, these authors attributed the observed changes to the formation of hydrogen bonds between nanocellulose and gelatin.

#### 3.2.5. X-Ray Diffraction

**Figure 6:**
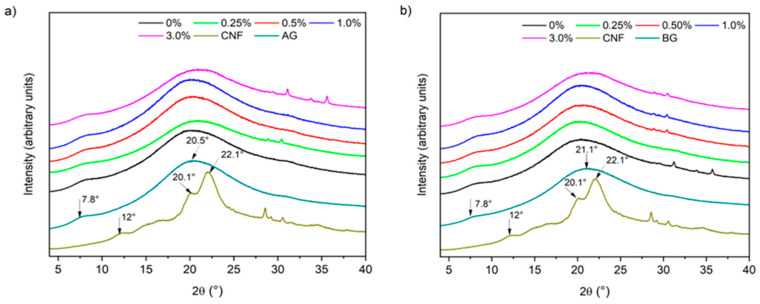
X-ray diffraction patterns of gelatin hydrogels containing cellulose nanofibrils: (**a**) type A and (**b**) type B gelatin.

Gelatin hydrogels showed similar diffraction patterns, regardless of the type of gelatin or the concentration of CNFs (Figure 6), because no modification on the gelatin peptides occurred, as observed by FTIR (Figure 5). These patterns were characterized by a broad amorphous peak observed at 2θ = 20°, which is related to the distance between amino acid residues along the gelatin chains [48]. A lower intensity peak was also observed at 2θ = 7.8°, characteristic of both AG and BG. Additionally, an increase in the CNF concentration did not change the spectra of these materials, as neither of the two characteristic peaks of this nanomaterial (2θ = 12 and 22°) was observed in the spectra of the hydrogels (Figure 5), probably due to the low concentrations studied. Thus, the presence of CNFs did not affect the crystallinity of these materials.

Ooi et al. [24] prepared glutaraldehyde-cross-linked gelatin hydrogels reinforced with rice husk CNCs (5–25%), and observed an increase in the crystallinity of the hydrogels only at concentrations > 5%. In turn, Rao et al. [48] studied the influence of amino-functionalized CNFs (5%) on hydrogels of gelatin cross-linked with genipin and were unable to distinguish the peaks of this nanomaterial in the spectra of the hydrogels. According to these authors, this may be related to the fact that large amounts of amorphous gelatin can mask the small amount of crystalline nanomaterial in the matrix. On the other hand, Dong et al. [25] did not observe peaks associated with CNCs (2%) in gelatin hydrogel reticulated by transglutaminase, behavior that was attributed to hydrogen bonds between CNCs and gelatin chains, as corroborated by mechanical properties, which did not allow the formation of regular crystallites in the hydrogel matrix. However, hydrogels containing CNFs did not evidence improvements in other properties that suggested the formation of hydrogen bonds. Therefore, the addition of CNFs probably did not change the hydrogel spectrum due to its low concentrations.

#### 3.2.6. Thermal Properties

**Figure 7:**
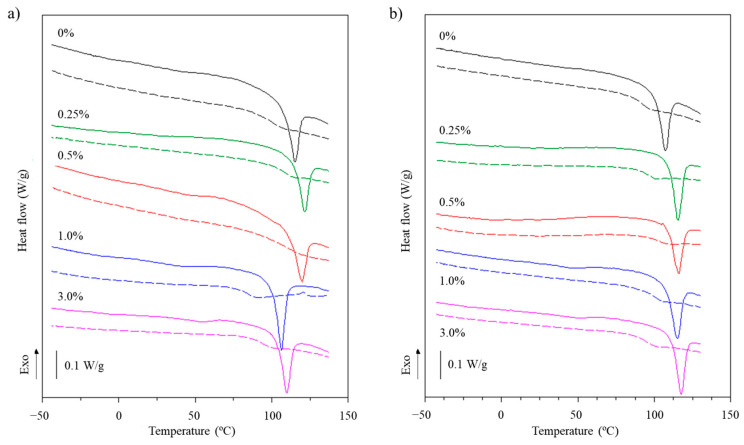
Heat flow curves of (**a**) type A and (**b**) type B gelatin hydrogels containing cellulose nanofibrils in different concentrations: solid lines—1st scan; dotted lines—2nd scan.

The heat flow curves of type A and B gelatin dried hydrogels containing cellulose nanofibrils in different concentrations are presented in the Figure 7.

In general, it was possible to observe that in the first scan, the dry hydrogels presented a glass transition (Tg) occurring at ~50 °C (Figure 7), which is clearly notable in the samples containing CNFs from soybean straw at a concentration of 3%. This phenomenon would correspond to a phase rich in some lower-molecular-weight fraction of gelatin. After this Tg, an endothermic peak appeared at ~110 °C (Table 2), which is associated with the melting of gelatin crystals. This behavior was typical of semicrystalline materials.

In the second scan, it was possible to observe only a Tg (~100 °C, Table 2), which was typical of a completely amorphous polymer [37]. The occurrence of this glass transition in the second scan must be associated with a higher-molecular-weight fraction of the gelatin. It must have occurred in the first scan, but was “eclipsed” by the melting of microcrystals, which involved much greater energy changes than the glass transition [37]. Similar observations were reported for freeze-dried AG hydrogels in the first scan [50] and in the second scan [51]. Regardless of the type of gelatin, the melting temperature of the hydrogels was not significantly affected (*p* > 0.05) by the incorporation of CNFs (Table 2), although an increasing tendency for type BG hydrogels was observed. Similar behavior was observed by Boughriba et al. [49], with gelatin hydrogels reinforced with microcrystalline cellulose.

The melting enthalpy, which reflects the energy required to melt the junction zones of the hydrogel network, exhibited an increase in concentration of only 1% for AGH (Table 2), suggesting greater thermostability of these hydrogels. In turn, the glass transition temperature of the second scan was not affected by the addition of CNFs at different concentrations either, suggesting that the presence of CNFs does not interfere with the mobility of the gelatin chains in their amorphous state. However, some studies on hydrogels suggest that the addition of nanomaterials into the hydrogel matrix improves thermal properties due to the establishment of stronger junction zones or an increase in the number of junction zones as reinforcement content increases [49]. In this study, the results of thermal properties may also be associated with the low CNF content present in gelatin hydrogels that did not induce homogeneous changes in hydrogel matrix. Overall, these results for thermal properties corroborate those observed by FTIR.

#### 3.2.7. Mechanical Properties

**Figure 8:**
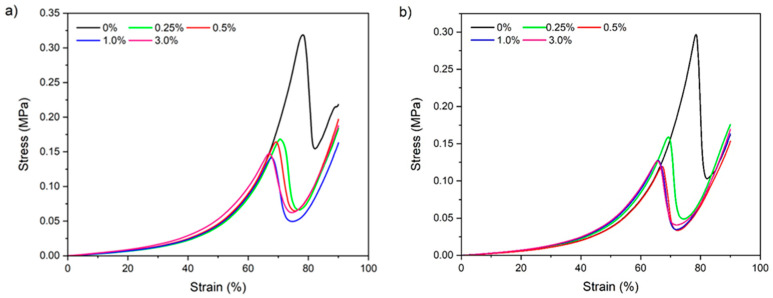
Deformation curves of (**a**) type A and (**b**) type B hydrogels and gelatin containing different concentrations of soybean straw cellulose nanofibrils.

Regarding the compression test results, all the samples showed similar behavior (Figure 8), with an elastic region, characterized by the linear portion of the curve (0–30%), corresponding to the linear viscoelasticity region, and a region in which the stress increased exponentially as a function of deformation, followed by fracture, characterized by the peak in the curves (Figure 8). The elastic modulus and the stress and strain at fracture were calculated with data from the first region and the fracture peak, respectively (Table 3).

An increase in the CNF concentration from 0 to 3% provoked a decrease in the fracture stress of the hydrogels by approximately 50%, regardless of the type of gelatin (Table 3). In other words, nanocelluloses did not improve the mechanical properties of the hydrogels, which may be related to the changes observed in the microstructures of the gels, as shown in Figure 3 and Figure 4, and the nature of this nanomaterial. The fracture deformation of the gelatin hydrogels also decreased with the addition of CNFs from 0 to 3%, being around 14% for both types of gelatins, suggesting the formation of less flexible structures with the addition of this nanomaterial. On the other hand, the elastic modulus of the hydrogels increased (*p* ≤ 0.05) at concentrations of 1% and 3% CNFs, suggesting that these materials can improve the mechanical properties of hydrogels in the elastic region. At the same time, these results evidenced that between the hydrogels and the CNFs, there are weak physical interactions.

Rao et al. [48] observed that the addition of amino-functionalized CNFs (5%) to physically cross-linked gelatin hydrogel did not significantly affect the compression properties of this material. However, after cross-linking with genipin (2%), hydrogels containing 0 to 5% amino-functionalized CNFs exhibited an increase of 0.84 to 1.52 MPa; indicating that in the presence of a cross-linking agent, CNFs can provide an improved ability to withstand large deformations. These authors attributed this behavior to the interactions of inter- and intramolecular covalent bonds formed within the hydrogels that allowed the load to be transferred efficiently across biopolymer matrix nanoparticles and to the high stiffness of the modified cellulose fibrils. However, Ge et al. [21] observed that the addition of chitin nanowhiskers (0–1%, *w*/*v*) to physical BG hydrogels slightly increased the fracture stress from 0.02 to 0.03 MPa, without changes in the fracture strain, increasing in the elastic modulus from 0.07 to 0.13 MPa. These improvements in mechanical properties were attributed to hydrogen interactions and electrostatic interactions between gelatin and chitin nanowhiskers.

Thus, the decrease in the compressive strength of hydrogels observed with the addition of soybean straw CNFs to the gelatin hydrogels could be related to the low degree of interfacial bonding between the nanomaterial and the gelatin matrix. In physical gelatin hydrogels, these interactions with CNFs depend mainly on the hydrogen bonds, which are weaker than covalent and ionic bonds, and in turn the large amount of water contained in the hydrogel networks could also have made it very difficult to interact between CNFs and gelatin polymer chains [48]. It is also necessary to clarify that during compression tests, the samples undergo high deformations that can destroy the fragile nanoparticle network [52] and that CNFs are less hydrolyzed materials than CNCs, thereby containing more heterogeneous structures and fewer hydroxyl groups on their surface, making it difficult to compare these results with the improvements in the mechanical properties of physical hydrogels observed with the addition of chitin nanocrystals produced by acid hydrolysis, as shown by Ge et al. [21]. These results also can be explained by the glass transition (Tg) of the material, which was not affected by CNFs, with the knowledge that mechanical properties of polymer systems are strongly dependent on its Tg.

In relation to the texture profile analyses, it was observed that increasing the nanomaterial concentration from 0 to 3% resulted in an increase in the hardness of the gelatin hydrogel of ~10% for AGH and ~25% for BGH (Table 4). Similar behavior was observed for gumminess and chewiness, which also increased from 458 to 505 g force and from 463 to 498 g force (AGH) and from 413 to 519 g force and from 463 to 498 g force (BGH), respectively. Similar observations were reported in AG hydrogels reinforced with carboxylated CNFs (0–7.5%, *w*/*w*) [22] and for BG hydrogels containing chitin nanowhiskers (0–1%, *w*/*v* of solution) [21]. The addition of these nanomaterials resulted in an increase in hardness (up to 150%), gumminess, and elasticity and suggested that the addition of rigid nanomaterials to the gelatin matrix led to the formation of a more compact three-dimensional network [21,22].

On the other hand, the elasticity and the cohesiveness of gelatin hydrogels did not vary as a function of increasing concentration (Table 4). The elasticity of the gelatin hydrogels was approximately 1, which indicated that these materials were highly elastic and that the addition of CNFs did not influence the elastic character of these materials. In turn, the cohesiveness of the gelatin hydrogels was approximately 0.97, indicating that there were no changes in the samples’ ability to resist the second compression as a function of concentration. In contrast, a decrease in the resilience of the hydrogels was observed as a function of concentration, which may indicate that the rebound capacity of the samples after the first compression was affected by CNFs. Similar behaviors were observed in gelatin hydrogels reinforced with carboxylated CNFs and chitin nanowhiskers for elasticity [22] and resilience [21].

The texture parameters showed that CNFs can improve the properties of gelatin hydrogels during chewing, although these improvements are very low when compared to those reported in the literature, probably due to the physical nature of these hydrogels. The variations observed in the texture parameters depending on the concentration can be attributed to the percolation of the CNFs, which may contain aggregates of different sizes and shapes, affected by the morphological characteristics of this nanomaterial [22]. Likewise, the formation of aggregates between the CNFs and the biopolymer chains may have contributed to the notable changes in pore size observed in the morphology of the hydrogels (Figure 3 and Figure 4).

#### 3.2.8. Viscoelastic Properties by Stress Relaxation Test

**Figure 9:**
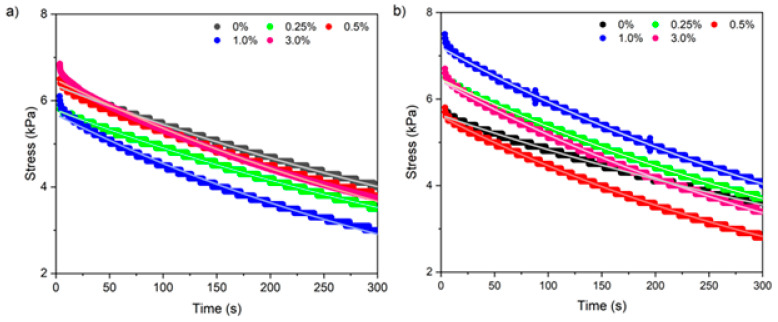
Stress relaxation curves of (**a**) type A and (**b**) type B gelatin hydrogels containing different concentrations of soybean straw cellulose nanofibrils. Markers: experimental data. Solid lines: Maxwell model.

Regardless of the type of gelatin and CNF concentration, all samples showed a behavior characterized by a decrease in stress from ~7 kPa to ~3 kPa as a function of analysis time (Figure 9), indicating that the hydrogels presented viscoelastic material behavior [34]. Similarly, Manish et al. [53] observed that when subjected to stress relaxation tests, hydrogels (10%, *w*/*v*) of gelatin cross-linked with glutaraldehyde at a humidity of 90% also showed viscoelastic material behavior. In turn, Bertsch et al. [33] reported that BG nanoparticle hydrogels showed exponential stress decay at strains greater than 20%.

The viscoelastic behavior of the hydrogels can be described by the Maxwell model (Equation (3)), which satisfactorily fit the experimental data (R^2^ = 0.99) (Table 5). The relaxation modulus (G) ranged from 28 to 32 kPa, while the viscosity (μ) ranged from 14 to 16 MPa·s^−1^ (Table 5). Thus, increasing the concentration of nanofibrils did not affect the viscoelastic properties of either type of hydrogel.

According to Manish et al. [53], the viscoelastic behavior of soft polymeric materials subjected to finite deformation can be attributed to chain entanglement mechanisms at the microscopic level. Specifically, at low strains, stress relaxation in gels occurred by rapid collective movements of percolated clusters or filaments on short scales and continuous structural rearrangements on long scales [33]. As a result, the presence of CNFs may have induced a non-uniform intertwining of the gelatin chains due to their aggregation. This makes increasing the relaxation modulus for both gelatin types difficult, regardless of the BG hydrogels’ evidenced highest values with the increase in concentration. In turn, the viscosity was not altered by the CNF content, indicating that at low deformations, due to its micro/nanometric scale dimensions, the gel did not flow. These viscosity values also show that there was no increase in attractive interactions within the CNF-containing hydrogels.

#### 3.2.9. Swelling and Water Retention Capacity

The hydrogels in the dry state had a moisture content between 4.8% and 6.1%, appearing as dry materials, without significant differences depending on the CNF content or the type of gelatin (Table 6). Increasing the CNF concentration (0–3%) decreased the swelling capacity of the hydrogels by ~38% for AGH and 32% for BGH (Table 6). These results agreed with those obtained by Ge et al. [21], who observed that the SW of BG hydrogels with the addition of 0 to 1% (*w*/*v*) chitosan nanowhiskers decreased from 152% to 126%, and by Ooi et al. [24], who observed that the addition of rice husk CNCs from 0 to 10% (*w*/*w*) provoked a decrease in SW from 700% to 617% at a pH close to neutrality.

On the other hand, the increase in the concentration of CNFs from soybean straw (0–3%) caused an increase in the WR of AGH (89.42% to 92.64%), while for BGH no increase was observed as a function of concentration (Table 6). This slight change observed in the WR of AGH could be related to the hydrophilic character of soybean straw CNFs, as a greater number of hydrophilic groups per unit volume in hydrogels can facilitate the retention capacity of water molecules [35]. Therefore, the results obtained with BGH may suggest greater interaction between this type of gelatin and CNFs, with fewer hydrophilic groups being available in these nanomaterials. However, according to Choudhury et al. [36], the ability of hydrogels to retain water decreases with increasing cross-linking of the biopolymer network.

According to Do Nascimento et al. [35], the SW and WR of hydrogels are closely related to the integrity of the network structure (pore size, morphology, and hydrophilic functional groups attached to its biopolymeric skeleton) and the extent of cross-linking. Thus, the results obtained suggest that the different concentrations of CNFs in soybean straw may not have contributed to the formation of a network with larger junction points. However, the results presented in Table 6 suggest that CNFs can be used as fillers to regulate the degree of swelling of gelatin hydrogels, which could be interesting for specific applications, such as tissue engineering, internal wound closure, soft actuating, and bioelectronics [54].

## 4. Conclusions

Physical and structural properties of hydrogels were regulated by electrostatic interactions between CNFs and type of gelatin. The application of CNFs as a reinforcing filler in hydrogel in a wet state was viable only at low deformations, mainly in relation to mechanical properties (elastic modulus, hardness, and chewability) without changes in its viscoelastic properties. These improvements were more remarkable in hydrogels based on type B gelatin. CNF concentration also influenced other surface properties, such as the color parameters and the opacity of hydrogels.

In dry-state hydrogels, the incorporation of CNFs induced the formation of a more heterogeneous and rigid network, which resulted in a decrease in swelling properties. The FTIR analysis and thermal properties of the hydrogels were not altered by the CNF concentration, suggesting a weak interfacial interaction between the CNFs and the gelatin, regardless of their type. This work evidence that the addition of CNFs as filler into complex matrices such as hydrogels depends on their morphology and compatibility with the matrix in terms of possible chemical or physical interactions. A greater range of concentrations of nanocellulose (nanocrystals or nanofibrils) into type B gelatin hydrogel must be further studied.

## Figures and Tables

**Table 1:** Color parameters and opacity of type A (AGH) and B (BGH) gelatin hydrogels containing cellulose nanofibrils at different concentrations (C_CNFs_).

Parameter	C_CNFs_ (%)	AGH	BGH
L*	0	86.5 ± 0.2 ^a.A^	85.7 ± 0.3 ^a.B^
	0.25	86.0 ± 0.1 ^a.A^	85.8 ± 0.2 ^a.A^
	0.5	85.9 ± 0.2 ^a.A^	85.7 ± 0.6 ^a.A^
	1	86.6 ± 0.5 ^a.A^	85.94 ± 0.8 ^a.A^
	3	85.5 ± 0.7 ^a.A^	83.63 ± 0.4 ^b.B^
a*	0	−0.8 ± 0.0 ^a.A^	−1.3 ± 0.0 ^a.B^
	0.25	−0.9 ± 0.0 ^b.a.A^	−1.3 ± 0.0 ^a.B^
	0.5	−0.9 ± 0.0 ^b.A^	−1.4 ± 0.0 ^a.B^
	1	−1.1 ± 0.0 ^c.A^	−1.5 ± 0.1 ^b.B^
	3	−1.5 ± 0.1 ^d.A^	−1.8 ± 0.0 ^c.B^
b*	0	4.2 ± 0.2 ^d.B^	8.8 ± 0.2 ^c.b.A^
	0.25	4.8 ± 0.1 ^d.c.B^	8.3 ± 0.1 ^c.A^
	0.5	5.1 ± 0.2 ^c.B^	8.8 ± 0.2 ^c.b.A^
	1	6.0 ± 0.2 ^b.B^	9.9 ± 0.8 ^b.A^
	3	7.4 ± 0.4 ^a.B^	12.9 ± 0.3 ^a.A^
∆E*	0	8.4 ± 0.3 ^b.B^	10.8 ± 0.3 ^c.b.A^
	0.25	9.0 ± 0.2 ^b.A^	9.2 ± 0.8 ^c.A^
	0.5	9.1 ± 0.3 ^b.a.B^	11.1± 0.4 ^c.b.A^
	1	9.3 ± 0.5 ^b.a.B^	11.6 ± 1.0 ^b.A^
	3	10.4 ± 0.8 ^a.B^	15.3 ± 0.5 ^a.A^
Opacity	0	2.4 ± 0.2 ^c.B^	3.3 ± 0.3 ^d.A^
	0.25	3.2 ± 0.2 ^c.b.B^	3.9 ± 0.2 ^c.d.A^
	0.5	3.2 ± 0.3 ^c.b.B^	4.4 ± 0.1 ^c.b.A^
	1	3.5 ± 0.3 ^b.B^	4.8 ± 0.1 ^b.A^
	3	6.6 ± 0.3 ^a.A^	7.1 ± 0.3 ^a.A^

Mean values ± standard deviation. Values in the same column with different lowercase letters and values in the same row with different uppercase letters differ significantly by Tukey’s test (*p* ≤ 0.05).

**Table 2:** Thermal properties of type A (AGH) and type B (BGH) gelatin hydrogels containing cellulose nanofibrils at different concentrations (C_CNFs_).

Parameters	C_CNFs_ (%)	AGH	BGH
T_m_ (°C) (I)	0	115.1 ± 0.3 ^a.A^	106.1 ± 6.0 ^a.A^
	0.25	115.7 ± 5.1 ^a.A^	111.3 ± 3.2 ^a.A^
	0.50	116.9 ± 4.2 ^a.A^	121.9 ± 7.7 ^a.A^
	1.00	108.7 ± 1.6 ^a.A^	117.7 ± 4.9 ^a.A^
	3.00	110.9 ± 5.2 ^a.A^	115.5 ± 1.4 ^a.A^
ΔH_m_ (J/g) (I)	0	17.6 ± 0.5 ^b.A^	17.7 ± 1.5 ^a.A^
	0.25	16.7 ± 0.5 ^b.A^	16.9 ± 1.0 ^a.A^
	0.50	17.7 ± 0.9 ^b.A^	16.4 ± 2.4 ^a.A^
	1.00	21.2 ± 1.5 ^a.A^	17.2 ± 0.6 ^a.B^
	3.00	17.3 ± 0.8 ^b.A^	17.1 ± 0.4 ^a.A^
T_g_ (°C) (II)	0	102.6 ± 8.3 ^a.A^	93.1 ± 4.8 ^a.A^
	0.25	107.2 ± 6.6 ^a.A^	95.3 ± 2.9 ^a.A^
	0.50	99.8 ± 7.7 ^a.A^	110.6 ± 12.4 ^a.A^
	1.00	90.4 ± 5.0 ^a.A^	102.7 ± 13.5 ^a.A^
	3.00	92.7 ± 6.2 ^a.A^	94.0 ± 2.2 ^a.A^

Mean values ± standard deviation. T_m_, melting temperature; ∆H_m_, enthalpy change; T_g_, glass transition temperature. (I) and (II), scans I and II. Values in the same column with different lowercase letters differ significantly by Tukey’s test (*p* ≤ 0.05). Values on the same line with different uppercase letters differ significantly by Tukey’s test (*p* ≤ 0.05).

**Table 3:** Mechanical compression properties of type A (AGH) and type B (BGH) gelatin hydrogels containing cellulose nanofibrils at different concentrations (C_CNFs_).

Properties	C_CNFs_ (%)	AGH	BGH
Fracture stress (kPa)	0	311.3 ± 10.5 ^a.A^	282.7 ± 12.9 ^a.A^
	0.25	169.0 ± 3.5 ^b.A^	161.0 ± 2.1 ^b.B^
	0.5	163.5 ± 1.7 ^c.b.A^	119.2 ± 2.3 ^c.B^
	1	144.7 ± 6.3 ^c.A^	129.8 ± 7.6 ^c.A^
	3	140.5 ± 9.6 ^c.A^	126.5 ± 0.3 ^c.A^
Fracture strain (%)	0	78.3 ± 0.4 ^a.A^	77.3 ± 0.6 ^a.A^
	0.25	70.8 ± 0.4 ^b.A^	69.5 ± 0.3 ^b.B^
	0.5	70.2 ± 0.5 ^b.A^	66.7 ± 0.4 ^c.B^
	1	68.6 ± 0.4 ^c.A^	66.4 ± 0.8 ^c.B^
	3	67.4 ± 0.5 ^c.A^	65.95 ± 0.5 ^c.A^
Elastic modulus (kPa/%)	0	0.30 ± 0.0 ^b.A^	0.28 ± 0.0 ^b.A^
	0.25	0.29 ± 0.0 ^b.A^	0.29 ± 0.0 ^b.a.A^
	0.5	0.30 ± 0.0 ^b.A^	0.28± 0.0 ^b.A^
	1	0.30 ± 0.0 ^b.a.A^	0.31 ± 0.0 ^b.a.A^
	3	0.36 ± 0.0 ^a.A^	0.34 ± 0.0 ^a.A^

Mean values ± standard deviation. Values in the same column with different lowercase letters differ significantly by Tukey’s test (*p* ≤ 0.05). Values in the same row with different uppercase letters differ significantly by Tukey’s test (*p* ≤ 0.05).

**Table 4:** Textural properties of type A (AGH) and type B (BGH) gelatin hydrogels containing cellulose nanofibrils at different concentrations (C_CNFs_).

Properties	C_CNFs_ (%)	AGH	BGH
Hardness (g force)	0	473.3 ± 8.8 ^b.A^	425.1 ± 5.4 ^c.B^
	0.25	469.7 ± 7.0 ^b.B^	488.8 ± 0.9 ^b.a.A^
	0.5	507.8 ± 14.6 ^a.A^	432.3 ± 17.3 ^b.c.B^
	1	463.4 ± 4.8 ^b.A^	508.2 ± 26.1 ^a.A^
	3	522.1 ± 8.8 ^a.A^	533.3 ± 23.1 ^a.A^
Springiness	0	1.00 ± 0.01 ^a.A^	0.99 ± 0.01 ^a.A^
	0.25	0.99 ± 0.00 ^b.a.A^	0.98 ± 0.01 ^a.A^
	0.5	0.98 ± 0.0 ^b.B^	0.99 ± 0.00 ^a.A^
	1	0.99 ± 0.00 ^b.a.A^	0.99 ± 0.01 ^a.A^
	3	0.99 ± 0.00 ^b.a.A^	0.99 ± 0.00 ^a.A^
Cohesiveness	0	0.97 ± 0.00 ^a.A^	0.97 ± 0.00 ^a.A^
	0.25	0.97 ± 0.00 ^a.A^	0.97 ± 0.00 ^a.A^
	0.5	0.96 ± 0.00 ^a.A^	0.97 ± 0.00 ^a.A^
	1	0.97 ± 0.00 ^a.A^	0.97 ± 0.00 ^a.A^
	3	0.97 ± 0.00 ^a.A^	0.97 ± 0.00 ^a.A^
Gumminess (g force)	0	458.1 ± 7.8 ^b.A^	413.3 ± 5.8 ^b.B^
	0.25	454.7 ± 6.1 ^b.B^	472.2 ± 3.0 ^b.a.A^
	0.5	490.6 ± 13.4 ^a.A^	419.8 ± 19.8 ^b.B^
	1	449.7 ± 5.2 ^b.A^	493.1 ± 25.8 ^a.A^
	3	505.0 ± 8.3 ^a.A^	518.9 ± 23.1 ^a.A^
Chewiness (g force)	0	462.8 ± 12.3 ^b.c.A^	413.4 ± 10.5 ^b.B^
	0.25	448.6 ± 4.9 ^c.A^	461.0± 7.8 ^b.a.A^
	0.5	478.4 ± 10.1 ^b.a.A^	423.1 ± 20.1 ^b.B^
	1	452.3 ± 1.6 ^b.c.B^	496.6 ± 15.0 ^a.A^
	3	497.9 ± 8.0 ^a.A^	513.50 ± 22.0 ^a.A^
Resilience	0	0.94 ± 0.00 ^a.A^	0.94 ± 0.00 ^a.A^
	0.25	0.93 ± 0.01 ^b.a.A^	0.91 ± 0.01 ^b.a.A^
	0.5	0.92 ± 0.01 ^b.c.A^	0.92 ± 0.00 ^b.a.A^
	1	0.93 ± 0.00 ^b.a.A^	0.91 ± 0.01 ^b.B^
	3	0.91 ± 0.00 ^c.B^	0.93 ± 0.00 ^b.a.A^

Mean values ± standard deviation. Values in the same column with different lowercase letters differ significantly by Tukey’s test (*p* ≤ 0.05). Values on the same line with different uppercase letters differ significantly by Tukey’s test (*p* ≤ 0.05).

**Table 5:** Relaxation modulus (G) and viscosity (μ) calculated by nonlinear regression of the Maxwell model of type A (AGH) and B (BGH) gelatin hydrogels containing cellulose nanofibrils at different concentrations (C_CNFs_).

Properties	C_CNFs_ (%)	AGH	BGH
G (kPa)	0	31.7 ± 0.6 ^a.A^	28.7 ± 1.4 ^a.A^
	0.25	28.7 ± 0.4 ^b.a.B^	32.1 ± 1.1 ^a.A^
	0.5	31.3 ± 0.9 ^a.A^	27.9 ± 0.9 ^a.B^
	1	28.0 ± 1.2 ^b.B^	31.10 ± 0.6 ^a.A^
	3	29.4 ± 1.4 ^b.a.A^	31.55 ± 2.8 ^a.A^
μ (×10^6^ Pa·s)	0	15.8 ± 0.3 ^a.A^	14.3 ± 0.7 ^a.A^
	0.25	14.3 ± 0.2 ^b.a.A^	16.0 ± 0.5 ^a.A^
	0.5	15.7 ± 0.4 ^a.A^	14.0 ± 0.5 ^a.B^
	1	14.0 ± 0.6 ^b.B^	15.6 ± 0.3 ^a.A^
	3	14.7 ± 0.7 ^b.a.A^	15.8 ± 1.4 ^a.A^
R^2^	0	0.998 ± 0.000	0.996 ± 0.001
	0.25	0.997 ± 0.000	0.998 ± 0.000
	0.5	0.996 ± 0.001	0.997 ± 0.001
	1	0.997 ± 0.000	0.998 ± 0.000
	3	0.998 ± 0.001	0.998 ± 0.000

Mean values ± standard deviation. R^2^, nonlinear regression correlation coefficient. Values in the same column with different lowercase letters differ significantly by Tukey’s test (*p* ≤ 0.05). Values on the same line with different uppercase letters differ significantly by Tukey’s test (*p* ≤ 0.05).

**Table 6:** Swelling (SW) and water retention capacity (WR) of type A (AGH) and type B (BGH) gelatin hydrogels containing cellulose nanofibrils at different concentrations (C_CNFs_).

Parameters	C_CNFs_ (%)	AGH	BGH
Moisture content (%)	0	6.0 ± 0.3 ^a.A^	5.3 ± 1.1 ^a.A^
	0.25	5.5 ± 0.7 ^a.A^	5.2 ± 0.1 ^a.A^
	0.5	5.8 ± 0.2 ^a.A^	4.8 ± 0.2 ^a.B^
	1	4.9 ± 0.7 ^a.A^	5.8 ± 0.5 ^a.A^
	3	6.1 ± 0.7 ^a.A^	5.1 ± 0.3 ^a.A^
SW (%)	0	2024 ± 33 ^a.A^	2023 ± 48 ^a.A^
	0.25	1456 ± 9.4 ^c.B^	1578 ± 52 ^b.A^
	0.5	1841 ± 43 ^b.A^	1473 ± 34 ^c.b.B^
	1	1428 ± 8.3 ^c.A^	1309 ± 57 ^d.B^
	3	1257 ± 47 ^d.A^	1370 ± 16 ^c.d.A^
WR (%)	0	89.4 ± 1.0 ^b.A^	91.2 ± 2.5 ^b.a.A^
	0.25	92.6 ± 0.4 ^a.A^	93.1 ± 0.8 ^a.A^
	0.5	89.8 ± 0.7 ^b.A^	85.8 ± 0.9 ^b.B^
	1	92.7 ± 1.1 ^a.A^	89.6 ± 1.9 ^b.a.A^
	3	92.6 ± 0.3 ^a.A^	91.9 ± 0.2 ^a.A^

Mean values ± standard deviation. Values in the same column with different lowercase letters differ significantly by Tukey’s test (*p* ≤ 0.05). Values on the same line with different uppercase letters differ significantly by Tukey’s test (*p* ≤ 0.05).

## Data Availability

The original contributions presented in this study are included in the article/Supplementary Materials. Further inquiries can be directed to the corresponding authors.

## Supplementary Materials

The following supporting information can be downloaded at https://www.mdpi.com/article/10.3390/foods14132269/s1. Figure S1. Soybean straw (a), milled soybean straw (b), and chemically treated soybean straw (c). Figure S2. Gelatin hydrogels containing nanofibers at concentrations of 0 (a, c) and 3% (b, d), and gelatin type A (a, b) and B (c, d).
