# Supplementary material for: Physical Properties of Gelatin-Based Hydrogels Incorporated with Soybean Straw Nanocellulose Obtained by Enzymatic Hydrolysis
Source: Foods. 2025 Jun 26;14(13):2269. doi: 10.3390/foods14132269 (PMC12248791; doi:10.3390/foods14132269)
Supplement: Supplementary file 1 [file foods-14-02269-s001.zip › foods-3687739-supplementary.pdf]

Physical properties of gelatin-based hydrogels incorporated with soybean straw nanocellulose obtained by enzymatic hydrolysis

Lía Ethel Velásquez-Castillo [1*], Gisele Imoto de Freitas [2], Izabel Cristina Freitas Moraes [2], Milena Martelli Tosi [2], Daniel Enrique López Angulo [3], Paulo José do Amaral Sobral [2,4]**

Supplementary materials

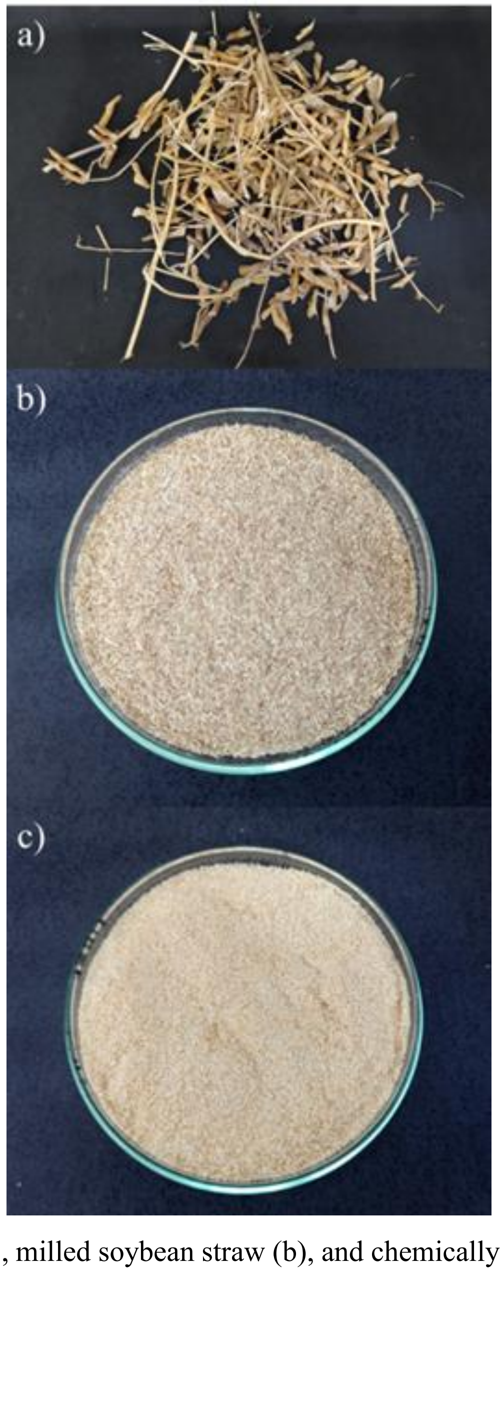

Fig. S1. Soybean straw (a), milled soybean straw (b), and chemically treated soybean straw (c).

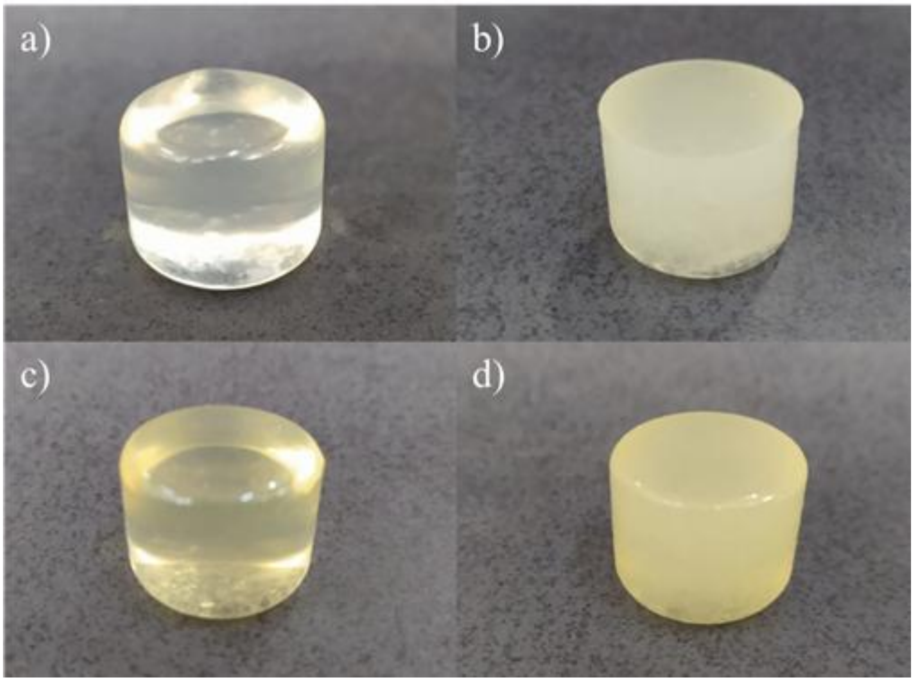

Fig. S2. Gelatin hydrogels containing nanofibers at concentrations of 0 (a, c) and 3% (b d), and gelatin type A (a, b) and B (c,d).
